# Analyzing Riemann-Liouville constraints in second-order Lagrangian fractional electrodynamic models

**DOI:** 10.1371/journal.pone.0320632

**Published:** 2025-05-27

**Authors:** Yazen M. Alawaideh, Bashar M. Al-khamiseh, Isaac Kwasi Adu

**Affiliations:** 1 MEU Research Unit, Middle East University, Amman, Jordan; 2 Mathematical Sciences Department, Kumasi Technical University, Kumasi, Ghana; Lahore School of Economics, PAKISTAN

## Abstract

This study used second-order fractional derivatives to constrain singular Lagrangians to construct comprehensive Hamilton-Dirac equations. Notable contributions include resolving the difficulties associated with fractional derivatives. This modeling methodology efficiently covers non-local and non-differentiable fractional derivatives, giving a systematic strategy for dealing with modeling complexity. It establishes fractional equations that connect Coulomb’s law to the principle of superposition. Furthermore, we extended the Hamilton-Jacobi formalism by incorporating second-order derivatives in the context of Podolsky’s electrodynamics. This approach provides a solution for overcoming limitations in singular Lagrangians by linking the principles of Lagrangian fractional electrodynamics and classical field theory. The novelty of this work lies in its methodological approach to resolving the challenges of second-order fractional derivatives, particularly with respect to non-locality and memory effects, which have not been adequately addressed in previous models. This research offers new insights into expanding classical field theory using fractional calculus, opening new avenues for understanding interactions in electrodynamic systems. The results suggest that fractional formulations broaden the boundaries of traditional theories, providing a framework that encompasses a wider range of dynamic behaviors and advancing the understanding of fractional electrodynamics beyond previous studies. Furthermore, this approach paves the way for future research into fractional special relativity theories.

## 1. Introduction

Fractional calculus is widely used in science and engineering [1–3] especially in physics and mechanics [4–12]. Researchers explore higher-order dynamical systems using methods like Dirac’s technique [13–15]. They use fractional Noether’s theorem and derivatives to study systems with higher-order derivatives [16–18]. Podolsky’s electrodynamics with second-order fractional derivatives is an example of fractional calculus [19]. In a recent study, a technique for systems with higher-order derivatives was introduced, deriving equations from the Degasperis-Procesi equation [20] as an example of second-order order. In another paper, researchers proposed a method for generating fields with fractional derivatives [21,22]. Other approaches calculate conserved variables using fractional Noether’s theorem [23], and Hamiltonian formalism with fractional derivatives is applied similarly to conventional mechanics [24]. Hamiltonian formalism has also been extended to classical fields with fractional derivatives, and its applicability has been demonstrated by solving two discrete problems and one continuous problem. The outcomes of these problems were consistent with Agrawal’s formalism. The work in [25] presented a simple numerical strategy to address the problems using the Riemann-Liouville fractional derivative. Two issues—time-invariant and time-varying—were solved using numerical solutions. The findings demonstrated that the solutions converged as the segment size increased in the time domain. The Riemann-Liouville fractional derivative is a comprehensive extension of ordinary calculus that encompasses most other fractional derivative definitions. It adheres to mathematical principles within fractional calculus. When used with Podolsky’s generalized electrodynamics, it provides the initial conditions with practical and mathematically sound derivatives. Caputo’s derivatives impose stricter regularity criteria and only apply to differentiable functions, while Riemann-Liouville fractional derivatives can extend to features without a first derivative. Researchers generally prefer the Riemann-Liouville definition over others, such as the Caputo or Atangana-Baleanu fractional derivatives. This method can be applied to Podolsky’s generalized electrodynamics model, which involves continuous systems with second-order fractional derivatives. The main contributions of this study are as follows: We noticed that there was perfect agreement between the Lagrangian formulation and the classical equations of motion that were obtained in our investigation. The advantage of using our formalism lies in the fact that the resulting Hamiltonian is linear in Pα, a characteristic that simplifies the analysis of the system and facilitates the application of fractional Hamiltonian mechanics. This linearity is particularly beneficial when dealing with systems governed by fractional derivatives, as it preserves the structure of the equations of motion while accommodating the non-local effects inherent in fractional calculus.

Fractional calculus has increasingly gained attention in various fields of science and engineering due to its ability to model non-locality and memory effects, which are not adequately captured by classical calculus methods. In electrodynamics, fractional derivatives, particularly the Riemann-Liouville formulation, offer a powerful tool for exploring complex interactions within systems characterized by non-local behavior and long-range interactions. For example, recent studies have successfully applied fractional calculus to address complex problems in fields such as fluid dynamics and electrodynamics [26,27].

In the context of fractional-order systems, there have been notable advances in both theoretical and practical aspects, such as the formulation of new models and the numerical methods used to solve them. For instance, a comparative study on fractional-order models for supercapacitors highlighted the effectiveness of fractional derivatives in capturing the behavior of time-varying systems [28]. Moreover, fractional electrodynamics has proven useful in extending classical theories to accommodate higher-order derivatives, which play a critical role in modern electrodynamics and quantum mechanical systems [29].

Podolsky’s electrodynamics, combined with fractional calculus, provides a promising framework for further exploring the dynamic behavior of electromagnetic fields, particularly under the influence of gravitational effects and other complex interactions [30,31]. This research seeks to extend the application of Riemann-Liouville fractional derivatives to second-order Lagrangian systems, further bridging the gap between classical and modern electrodynamics. The findings from recent studies have reinforced the importance of developing models that capture the intricacies of fractional systems, especially those that involve non-locality and memory [32,33]. In addition to these advancements, researchers have also explored the application of fractional calculus in nonlinear dynamical systems, such as the Hyperbolic Lane–Emden System [34], which extends the classical Lane–Emden equation to describe time-dependent phenomena in astrophysics and plasma physics. Similarly, the Coupled Lane–Emden–Klein–Gordon–Fock system with central symmetry [35] has been studied to understand the symmetries and conservation laws in systems combining Lane–Emden and Klein–Gordon–Fock equations, which are crucial in relativistic quantum mechanics. Furthermore, the Reductions and exact solutions of the (2+1)-dimensional breaking soliton equation [36] demonstrate how conservation laws can be used to derive exact solutions for nonlinear partial differential equations, providing insights into the behavior of solitons in higher-dimensional systems. These studies highlight the versatility of fractional calculus and its ability to address complex nonlinear phenomena across various scientific domains.

We investigated the second-order Lagrangian of systems with fractional derivatives to test our formalism and gain a better understanding. This conclusion was found to be in perfect agreement with the Hamiltonian model. These experiments were intended to pave the way for the eventual development of fractional relativistic special relativity. The use of the Riemann-Liouville function in the generic fractional Lagrangian technique provides a more adaptable model than classical modeling. This property can be used to explain the relationship between Dirac theory in ordinary Podolsky theory and higher-order field theories, typical fractional formulations, and restricted systems. Consequently, we believe that the Podolsky theory, in general, and the solution technique outlined in this study are innovative and encompass information that is markedly different from that contained in comparable standard classical equations. The RL fractional derivative, also known as the Riemann-Liouville fractional derivative, is popular in fractional calculus due to its simplicity and connection to the theoretical foundations. It offers a more thorough depiction of system behavior and depicts non-locality in fractional systems, such as fractional electrodynamics. RL fractional derivatives take into account both the past and present, in contrast to Caputo fractional derivatives, which permit non-locality modeling and long-range interactions. Taking into account the benefits and drawbacks of each operator, the fractional derivative operator selected will rely on the particular situation at hand as well as the level of modeling accuracy that is required. The goal of this work is to extend Dirac’s theory of restricted systems to higher-order field theories and provide a thorough examination of fractional Podolsky theory. The goal is to clarify the interactions between these two frameworks and investigate their ramifications in a more in-depth and thorough way.

This study presents a novel approach to addressing challenges posed by fractional derivatives in second-order Lagrangian systems, particularly in electrodynamics, by extending Riemann-Liouville fractional derivatives. It offers new insights into non-local behavior and memory effects, which have been overlooked in previous research, bridging fractional calculus with classical field theory. The integration of second-order fractional derivatives with classical electrodynamics overcomes singular Lagrangian limitations and extends Podolsky’s electrodynamics, broadening its applicability and laying the foundation for future research in fractional special relativity. This contribution demonstrates the advantages of fractional calculus in capturing complex real-world phenomena.

The remainder of this manuscript is organized as follows: In Section 2, we provide a brief overview of the definitions of fractional derivatives. Section 3 focuses on Podolsky’s fractional special relativity equation. In Section 4, we discuss second-order singular Lagrangian fractional constraints. Section 5 explores various applications of fractional calculus and suggests potential directions for future research. Section 6 presents a comparison of our results with those from other studies, highlighting both similarities and differences. Finally, Section 7 concludes the paper with some final remarks.

## 2. Overview of properties and definitions for Riemann-Liouville fractional derivative

In this section of our study, we will provide a brief overview of the fundamental properties and definitions that are used throughout this work. Specifically, we will be discussing the Riemann-Liouville derivative, which is also referred to as the fractional derivative. Its definition, as given in [37], is presented below:


Dxαa f(x)=1Γ(n−α)(ddx)n∫axf(τ)(x−τ)α−n+1dτ.  (1)

The right Riemann- Liouville fractional derivative


Dbαx f(x)=1Γ(n−α)(−ddx)n∫xbf(τ)(x−τ)α−n+1dτ.   (2)

where Γ denotes the Gamma function, and  α   is the order of the derivative such that n− 1< α< n. If α is an integer, these derivatives are defined in the usual sense, i.e.,


Dxαa f(x)=(ddx)nf(x)

Dtαa f(x)=(ddx)nf(t)            α=1,2,.  

The Riemann-Liouville fractional derivative is a generalization of the ordinary derivative to non-integer orders. Here are some properties of the Riemann-Liouville fractional derivative:

1. Fractional Linearity: The fractional derivative, denoted by $D^{\alpha }$, follows the property of fractional linearity. For any real numbers α and β and functions f(x) and g(x), we have:

Dtαa (af(x)+bg(x))=aDtαa (f(x))+bDtαa (g(x)).

1. Commutativity: The fractional derivative commutes with respect to the order of differentiation. In other words, for any two real numbers α and β, we have:

Dtαa (Dtαa (f(x)))=Dtαa (Dtαa (f(x))).

1. Fractional Integration: The Riemann-Liouville fractional derivative is related to fractional integration. The fractional integral operator Itαa  of a function f(x) is defined as the inverse of the fractional derivative Dtαa . That is, for any real number α, we have:

Itαa (Dtαa (f(x)))=f(x).

1. Homogeneity Property: The fractional derivative has a homogeneity property, which means that for any real number α and a constant c, we have:

Dtαa (cf(x))=cDtαa (f(x)).

These are some of the key properties associated with the Riemann-Liouville fractional derivative.

## 3. Podolsky’s fractional special relativity equation

When the electric charge is assumed to be a globally conserved scalar, charge density transforms as the zero component of a four-vector. This 4-vector represents the current density $j^{\mu }\left(j^{0},j^{\to }\right)$where $j^{0}=\rho$. Global charge conservation implies local charge conservation. The continuity equation for electric charge is obtained.


DaxαjμμA=0

According to the assertion, i can have values of 1, 2, and 3 when μ is equal to zero. That holds true for all Lorentz referential as well. To make the equation more widely applicable.


(1−μ2Daxαμ)Daxα.E―(r―)=ρ(r―)

To its Lorentz-transformed shape-variant form, we need to account for the effects of relativity. To put it another way, the equation must be expressed in a fashion that complies with the rules of physics in every inertial frame of reference, independent of the frames relative motion. Here a mathematical technique known as the Lorentz transformation aids in the conversion between several reference frames that are moving in relation to one another at a constant velocity. It enables us to take into consideration the impacts of relativistic phenomena like length contraction and time dilation that may have an impact on how physical systems behave. The charge density can then be expressed in terms of J after the equation has been generalized to its Lorentz-transformed shape-variant form. This is thus because the charge density in the rest frame of the charge distribution is represented by J, a scalar function. We may then use J to compute the charge density in any frame of reference, independent of its relative speed, by formulating the equation in a way that is consistent with the laws of physics as they apply in all inertial frames of reference.


(1−μ2Daxαμ )DaxαEi=j0 (3)

Davα(Daxαμ Av−Daxαv Aμ\) is the zero component of a four-vector if (Daxαμ Av−DaXαv Aμ\) is a second-order tensor. It is not difficult to demonstrate that the electric field $E^{i}$ may be represented by the second-order tensor component shown below. if *i*=1,2,3. Because the right side of (3) is the zero component of a 4-vector, we obtain by the following second-order tensor components:


Fμν=Daxαμ Av−DaXαv Aμ (4)

where, μ=0 , i=1,2,3 , v=0 , j=1,2,3.

Take μ=0, v=1 as an example:


$$F^{01}=\partial^{0}A^{1}-\partial^{1}A^{0}=E^{1}$$

Similarly, if we repeat the processes for Equation (5), we get:


$$\left(F^{01}=E^{2},\;F^{02}=E^{2},F^{03}=E^{3}\right)$$ (5)

Using the method of mathematical induction with the aid of Equation (23), it can be concluded that Equation (22) implies the following statement.


(1+a2πijDaXαi DaXαj )DaXαv F0v=j0 (6)

The term $\pi^{ij}$ “metric versus variant tensor” refers to the special relativity tensor, which in our usage contains the following elements:

The phrase “metric versus variant tensor” refers to a tensor in special relativity that is denoted as $\pi^{ij}$ and has specific components. It is used to distinguish between two types of tensors in relativity: metric tensors and variant tensors. The components of this tensor can be described in a different manner to help understand its characteristics, such as symmetry, positive definiteness, and nondegeneracy.


$$\pi^{00}=-\pi^{11}=-\pi^{22}={-\;\pi }^{33}=1.$$

Consequently, all sides of (6) change into the zero component of a 4-vector. As a result of generalizing Lorentz’s laws to include all 4-vectors and making them form invariant Lorentz transformations (6), we get:


(1+a2πμvDaxαμ DaXαv )DaXαv Fμv=jμ (7)

Taking the fractional derivative (Daxαμ \) of Eq. (6) now yields:


(1+a2πμvDaxαμ DaXαv )Daxαμ DaXαv Fμv=0 (8)

Equation (8) is said to be antisymmetric. As a consequence of the previous statement, Equation (7) is zero. This is the most direct solution we will use. Moving on, we are asked to choose the last three components from the equation Fμν=Daxαμ Av−DaXαv Aμ. To accomplish this, we can rewrite the equation as:


$$F^{\mu \nu }=\partial^{\mu }A^{v}-\partial^{v}A^{\mu }.$$

Here, we have used the notation $\partial^{\mu }$ to represent the partial derivative with respect to the μ th coordinate. Now, we can select the last three components by replacing  μ and v with the desired coordinates:


$$\left\{ \begin{array}{c} F_{23}=\partial_{2}A_{3}-\partial_{3}A_{2}.\\ F_{13}=\partial_{1}A_{3}-\partial_{3}A_{1}.\\ F_{12}=\partial_{1}A_{2}-\partial_{2}A_{1}. \end{array}\right.\;$$

These equations represent the last three components of the electromagnetic field strength tensor, which are often referred to as the “magnetic field” components.

Take as an example μ=i, ν=2, α=1,


$$F^{i2}=\partial^{i}A^{2}-\partial^{2}A^{i}={\;B}^{3}$$

Similarly, if we use the same techniques to solve equation Fμν=Daxαμ Av−DaXαv Aμ, *we get:*


$$F^{i3}={-B}^{3},\;F^{23=}{\;B}^{i}.$$

The magnetic induction field is represented by $\left(\vec{B}=B^{1},B^{2},B^{3}\right)$. One of the equations in Podolsky’s pair is the left (A4) equation, which is the relativistic version of the left (A4) equation (see Appendix in S1 File). Use generalization to find the missing equation (A2). If i, j, and k are an even permutation (odd), the${\;\varepsilon }^{ijk}$ Levi-Civita symbol ijk = +1 (-1) and the 1, 2, and 3 cancel out if any two indices are equal.


εijkDaXαj (DaXαk A0−Dtαa Ak)=0 (9)

where


(DaXαk A0−Dtαa Ak)=−(DaXαk A0−Dtαa Ak)=(Dtαa Ak−DaXαk A0)

The standard equations for integer order are generated if the above equation has an if statement of α=1. These are listed in the following order:


$$F^{0k}={-F}^{0k}=F_{k0}=E^{k}$$

The fourth-order totally antisymmetric tensor, represented by the symbol $\varepsilon^{\mu v\rho \lambda }$, is a mathematical tool that can transform equations. When its determinant value $\varepsilon^{0123}$ is evaluated, the result is **+1**. Using its components $\varepsilon^{ijk0}$ (which is equivalent to $\varepsilon^{ijk}$), the tensor can transform Equation (8) and simplify mathematical problems.


εijk0DaXαj (DaXαk A0−Dtαa Ak)=0 (10)

After establishing Eq. (9) invariance under the Lorentz transformation, we may now explore Einstein’s general theory of relativity. As a result, it is evident that, εμvρλDaXαv (DaXαρ Aλ−DaXαλ Aρ)=0 the covariant metric tensor (Daxαα Aβ−Daxαβ Aα) of special relativity; $\eta_{\alpha \mu }$ its covariant tensor is denoted by the formula


(Daxαα Aβ−Daxαβ Aα)=ηαμηβv(Daxαμ Av−DaXαv Aμ).

## 4. Second-order singular Lagrangian fractional restrictions

The essential features of the generalized electrodynamics in [38] suggested will be discussed here. On the Lagrangian, it is based.


$${ℒ}_{LW}=\;-\;\frac{1}{4}F_{\mu \nu }F^{\mu \nu }-\;a^{2}\partial_{\lambda }{F^{\alpha \lambda }\partial }^{\alpha }F_{\rho \alpha }.$$ (11)

where $F_{\mu \nu }=\partial_{\mu }A_{v}-\partial_{v}A_{\mu }$

So, we can write the Lagrangian (11) as:


$$L=-\;\frac{1}{2}F_{0i}F^{0i}-\;a^{2}\left[{\left(\partial_{i}F^{0i}\right)}^{2}-{\left(\partial_{0}F^{0i}\right)}^{2}\right].$$

To recreate the Podolsky Lagrangian density in Riemann-Liouville fractional form, we use the following relations:


[Fμν=Daxαμ Av−DaXαv AμFμν=Daxαμ Av−DaXαv Aμ]

[∂α=Daxαμ =(Dtαa ,DaXαi )∂α=Daxαμ =(Dtαa ,−aDxiα)]

FμνFμν=2[Daxαμ AvDaxαμ Av−Daxαμ AvDaXαv Aμ]

$$\left[\begin{array}{c} A^{\alpha }=\left(\phi ,\vec{A}\right)\\ A_{\alpha }=\left(\phi ,-\vec{A}\right) \end{array}\right]$$

The fractional electromagnetic lagrangian density formulation is as follows:


L=− 12[(Dtαa Ai)2−2Dtαa AiDaXαi ϕ+(DaXαi ϕ)2]−a2[[DaXαi Dtαa Ai−Dtαa DaXαi ϕ]2−[Dtαa DaXαi Ai−DaXαi Dtαa ϕ]2]      

The expression you provided relates the field strength tensor $F^{\mu \nu }$ to the potentials $A^{\mu }$ and $A^{v}$ through the covariant derivative operator D. The indices μ and ν represent spacetime indices, while α and a represent internal indices associated with the gauge symmetry group. The constant and has dimensions of length raised to the power of a.

When a=0, the Lagrangian generates a U(1) gauge theory, which is a linear field theory with gauge symmetry. This theory reduces to the familiar Maxwell theory, which describes the electromagnetic field, in the limit where the fields are weak and the electric charge is small. In this limit, the field strength tensor $F^{\mu \nu }$ reduces to the familiar expression $\;F^{\mu \nu }=\partial^{\mu }A^{v}-\partial^{v}A^{\mu }$, which relates the field strength to the vector potential Aμ.

In summary, the expression you provided relates the field strength tensor Fμν to the potentials $A^{\mu }$ and $A^{v}$ through the covariant derivative operator D. When a=0, the Lagrangian generates a U(1) gauge theory, which reduces to Maxwell theory in the weak-field limit.

These are the motion-related Euler-Lagrange equations.


[1+2a2((Dtαa )2−(DaXαi )2)][Dtαa +DaXαi ][DaXαi φ−Dtαa Ai]=0 (12)

If α it goes to 1, then Eq. (12) becomes:


$$\left(1-2a^{2}\;\right)\partial_{\lambda }F^{\lambda \alpha }=0$$

In which $\begin{array}{c} {\;\eta }^{\mu \nu }\partial_{\mu }\partial_{v}=\left(\partial_{0}^{2}-\nabla^{2}\right). \end{array}$ According to the potentials, the aforementioned equations read


(1−2a2 ημνDaxαμ DaXαv )Daxαμ DaXαv Aα−∂(1−2a2 ημνDaxαμ DaXαv )∂βAβ=0

The Lagrangian has the following expression:


Ei=Dtαa Ai−Diαa At,

where E represents the magnetic field and $B^{i}$ is the electric field, given by $\;B^{i}=\frac{1}{2}\varepsilon^{ijk}$ Dtαa Aj−Diαa Ak.

In summary, the above equation defines the Lagrangian in terms of the magnetic and electric fields, as well as the vector potential $A_{i}$ and $A_{t}$. An alternative expression for the Lagrangian is


ℒ=12((E→)2−(B→)2)+a2[(DaXαi .E→)2−(Dtαa E→−DaXαi ×B→)2

The motion equations are formulated as follows:


(1−2a2 ημνDaxαμ DaXαv )DaXαi .E→=0.

(1−2a2 ημνDaxαμ DaXαv )(E→−DaXαi ×B→)=0.

The energy-momentum tensor contains the following formula according to Poisson bracket formalism:


Tμv=−Fμα(DaXαv Aα−Daxαα Av)+14ημν(Daxαα Aβ−Daxαα Aα)Fαβ+a2ημν[DaXαμ Fμαμ λ Fαβ+FαβημνDaXαμ DaXαv Fαβ]−2a2[FμαημνDaXαμ DaXαv Fvα+FvαημνDaXαμ DaXαv Fμα+Daxαα Fμαμ β FvB]. (13)

The energy-momentum tensor can be constructed simple to demonstrate that it satisfies the conservation equation, which is given by Daxαα  or (Daxαα Tμv=Fβvjα) in the presence of external sources represented by the current $j^{\alpha }$. However, it should be noted that the energy-momentum tensor $T_{\mu v}$ does have a trace component, which means that the sum of its diagonal elements is not necessarily zero.


T=−2a2Daxαα (Daxαβ Aα−Daxαα Aβ)FαβDaXαλ FvB.

The absence of conformal invariance in the theory is intimately linked to the behavior of the potentials $A_{\mu }$(x). These potentials do not exhibit behavior that is entirely consistent with that of massless fields, as indicated by Equation (13) and the related comments. Equation (12) confirms the existence of this deviation.


T00=12((E→)2+(B→)2)+a2[−(Dtαa E→−DaXαi ×B→)2+2(E→.ημνDaxαμ DaXαv E→+B→.ημνDaxαμ DaXαv B→)+(DaXαi .E→)2].    (14)

In general, the tensor $T_{\alpha \beta }$, defined as the energy-momentum tensor in the theory of relativity, may not be positive definite. However, in the specific case of electrostatics, with the fields Dtαa E→ and $\vec{B}$, it can be shown that $T_{00}$ is positive. Assuming that $\vec{E}$ approaches zero faster than **1/r** at infinity, we can obtain an expression for the total energy E, which is given by integrating $T_{00}$ overall space. This expression can be rewritten as:


E=∫d3xT00=∫d3x[E→2+a2(DaXαi .E→)2] (15)

where a is a constant. For a point charge the electrostatic potential is given by


$$\emptyset (r)=\frac{e}{r}\left(1-e^{\frac{r}{a\sqrt{2}}}\right)$$ (16)

It is important to note that this result makes it straightforward to show that the total energy, as stated in Equation (15), has a finite value and equals $\frac{e^{2}}{2a}$ The calculation for ϕ(r) presented above should also take into account a Yukawa-type potential with a mass parameter of $\frac{1}{a\sqrt{2}}\;$ in addition to the standard Coulomb potential.

We note that the Lagrangian is singular with respect to the Hamiltonian formalism.


Hαβ∂2ℒDaX2α(∂Aα)DaX2α(∂Aβ)=−δα0δβ0+δαβ.

$$\det\left(H_{\alpha \beta }\right)=0.$$

The Lagrangian density of a continuous system is based on the second-order derivatives of the variables in the dynamical field, represented by L=L(ψ,Dtαa ψ,DaX2αψ). The indices used are Greek (0–3) and Latin (1–3), with the measure $\eta^{\mu \nu }=diag\;(+\;1,\;-\;1,\;-\;1,\;-\;1,\;-\;1)$ for both. To accommodate the Euler-Lagrange equations of motion generated by this Lagrangian, the Podolsky Lagrangian formalism must be extended.


∂ℒ∂ψρ−Daxαμ ∂ℒ∂(Daxαμ ψρ)+Daxαμ DaXαv ∂ℒ∂(Daxαμ DaXαv ψρ)=0

And that the momenta, conjugated, respectively, to $p_{a}$ and $\pi_{a}$, are


pa=∂ℒ∂Dtαa ψρ−2∂k∂ℒ∂(∂kDtαa ψρ)−∂0∂ℒ∂(DaX2αψρ)=−(Dtαa Aμ−Daxαμ A0)−2a2(DaXαk DaXαλ F0λδμk−Dtαa DaXαλ Fμλ) (17)

πa=∂ℒ∂(DaX2αψρ)= 2a2(DaXαλ F0λδμ0−Dtαa DaXαλ Fμλ).

If we write the aforementioned expression using the definitions of $p_{a}$ and $\pi_{a}$, we obtain the following:


p→=−E→−2a2[DaX2αE→−DaXαi ×B→−DaXαi (DaXαi .E→)]

π→=2a2[Dtαa E→−DaXαi ×B→].

The primary constraints are $\phi_{1}=\pi_{0},$ and ϕ2=p0−DaXαk πk=0.

Applying the definition of **π**, we can express the accelerations of point $\overset{―}{A\;}$ with respect to the frame of fractional form D as Dtαa  $\overset{―}{A}$.


Dtαa A―=12a2(πi)+DaXαk (Fik)+DaXαi A―0 (18)

The canonical Hamiltonian is defined as follows:


Hc=∫d3x(Pα1A―μ+πμDtαa A―μ−L).

Using equation above as a basis, we can arrive at the following:


Hc=∫[A―0DaXαi πi+PiA―i+12a2(πi)(πi)+πiDaXαk (DaXαi Ak−DaXαk Ai)+πiDaXαi A―0+14(Fμv)(Daxαμ Av−DaXαv Aμ)+12a2(A―i−DaXαi A0)(A―i−DaXαi A0)−a2(DaXαk A―k−DaXαk DaXαk A―0)(DaXαi A―i−DaXαi DaXαi A0)]d3x.

To obtain the canonical Hamiltonian by Dirac’s method, we add an arbitrary linear combination of the principal constraints to the existing Hamiltonian. This result in the equation $H_{T}=H_{c}+\int \left(C_{1}(xlambda_{1}+C_{2}(x)\lambda_{2}\right)d^{3}x$

Since c = 1, 2 and Dtαa λc={λc,HT}≈0,

We have the consistency conditions Dtαa λ1={λ1,HT}≈0


Dtαa λ2={λ2,HT}=DaXαk Pk≈0

where a is an arbitrary constant. This leads to a second constraint.


λ3=DaXαk Pk≈0. (19)

It is important to note that these constraints are of the highest caliber and are the only ones that can be verified easily. Finally, the extended Hamiltonian is expressed as:


$$H_{T}=H_{c}+\int \left(C_{1}(x)\lambda_{1}+C_{2}(x)\lambda_{2}\right)d^{3}x.$$

The Hamiltonian is responsible for the system’s temporal evolution and complete gauge freedom.

The dynamical variables’ equations of motion, given by Dtαa Aα={Aα,HE}, are as follows:


Dtαa A0=A―0+C2 ;Dtαa Ai=A―i−DaXαi C3. (20)

Simply put, ${\overset{―}{A}}^{\alpha }$ equals Dtαa Aα plus additive arbitrary functions. In addition, Dtαa A0={Aα,HE} gives


Dtαa A―0=C;Dtαa A―i=12a2(πi)+DaXαk (DaXαi Ak−DaXαk Ai)+DaXαi A―0 (21)

As a result, both ${\overset{―}{A}}^{0}$ and $A^{0}$ are arbitrary, while we have again obtained Equation (18).

The subject at hand is the study of the Hamiltonian equations of motion. First, we see that the Hamiltonian in question exhibits the property of being linear in $p_{\alpha }$, which is a property of the Hamiltonian approach for second-order systems. As a result, the equations of motion for $A_{\beta }$ essentially tell us that, from a Hamiltonian perspective, $\overset{―}{A_{\beta }}\;$ is defined in terms of Dtαa Aβ up to an additive arbitrary function. However, the equations of motion for A(x) reproduce equations Dtαa  A―=12a2(πi)+DaXαk (Fik)+DaXαi A―0, whereas we obtain DaXαi A―0 = C for $\overset{―}{A_{0}}$). This suggests that both ${\overset{―}{A}}_{0}$ and${\;A}^{0}$ are arbitrary functions.

In the case of the momenta variables Datαπi={πi,HE} and DatαPα={Pα,HE}, the following results are obtained:


Dtαa πi=−(Dtαa Ai−DaXαi A0)−2a2DaXαi DaXαk (Dtαa Ak−DaXαk A0)−Pi (22)

Dtαa p0=−(Dtαa Ai−DaXαi A0)−2a2DaXαi DaXαi DaXαk (Dtαa Ak−DaXαk A0) (23)

Dtαa pi=−DaXαi DaXαk πk+DaXαk DaXαk πi−DaXαk (DaXαi Ak−DaXαk Ai) (24)

Equation (22) is the concept of $p_{i}$ given by Equation (17), which when combined with (19) yields constraint $\lambda_{3}$. The demonstration that the gauge transformations in the theory are generated by the constraint’s equations can be performed using the approach detailed in [39]. The point of origin for the generator is as follows:


$$\tau =\int d^{3}x\left(\gamma_{a}{\left(\varrho_{a}\right)}_{p}+\Gamma_{b}{\left(\varrho_{b}\right)}_{s}\right).$$

The first-class (main) limitations are ${\left(\varrho_{a}\right)}_{p}$ and ${\left(\varrho_{b}\right)}_{s}$, respectively. Two coefficients, $\gamma_{a}$ and $\Gamma_{b}$, are presented in a differential equation.


Dtαa Γa+ΓbAba+γaBba=0.

Where $\left\{{\left(\varrho_{s}\right)}_{p},H_{E}\right\}={A_{ba}\left(\varrho_{b}\right)}_{s},\;\left\{{\left(\varrho_{a}\right)}_{p},H_{E}\right\}={B_{ab}\left(\varrho_{s}\right)}_{b},$

In our case the resulting generator is


τ=∫d3x(pμDaxαμ Γb+πμDaxαμ σ)

σ=Dtαa Γ, it follows that


δAα={Aα,τ}=Daxαμ Γ,  δpα=0

δA―α=Daxαμ σ,  δπα=0.

Consequently, the theory’s gauge transformations produced by (τ=∫d3x(pμDaxαμ Γb+πμDaxαμ σ)) are correct.

Using Hamilton-Jacobi formalism, we can derive the following:


$$H_{0}^{\prime }=H_{C}+P_{0}\;;\;P_{0}=\frac{\partial S\;}{\partial t\;}$$

H1′=π0;H2′=P0−DaXαk  πk.

In the case of $A^{i},$ the total differential equation is:


$${dA}^{i}=\frac{\partial H_{0}^{\prime }}{\partial p_{i}}dt+\frac{\partial H_{1}^{\prime }}{\partial p_{i}}d{\overset{―}{A}}_{0}+\frac{\partial H_{2}^{\prime }}{\partial p_{i}}dA_{0},$$

$${dA}^{i}=\frac{\partial H_{0}^{\prime }}{\partial p_{i}}dt=\frac{\partial H_{c}^{\prime }}{\partial p_{i}}dt.\;\text{Then}\text{ the Equation Above will take the form }{dA}^{i}={\overset{―}{A}}^{i}dt.\mathrm{\backslash ]}$$

Since C3 is arbitrary, it is completely equivalent to Equation (21). With respect to ${\overset{―}{A}}^{i}$, the total differential equation is:


$${d\overset{―}{A}}^{i}=\frac{\partial H_{0}^{\prime }}{\partial p_{i}}dt+\frac{\partial H_{1}^{\prime }}{\partial p_{i}}d{\overset{―}{A}}_{0}+\frac{\partial H_{2}^{\prime }}{\partial p_{i}}dA_{0},$$

dA―i=(12a2(πi)+DaXαk (Fik)+DaXαi A―0)dt.

The fractional form of the above equation is the same as the fractional form of the equation obtained from Dirac’s method. $p_{i}$ and $p_{0}$ can be determined as follows:


$${dp}^{i}=-\frac{\partial H_{0}^{\prime }}{\partial A_{i}}dt-\frac{\partial H_{1}^{\prime }}{\partial A_{i}}d{\overset{―}{A}}_{0}-\frac{\partial H_{2}^{\prime }}{\partial A_{i}}dA_{0}=-\frac{\partial H_{0}^{\prime }}{\partial A_{i}}dt=-\frac{\partial H_{c}}{\partial A_{i}}dt$$

dpi=−∫d3x[πjDaXαk (∂(DaXαi Ak−DaXαk Ai)∂Ai)−12(DaXαj An−DaXαn Aj)∂(Fjn)∂Ai]dt                     

dpi=[−DaXαi DaXαk πk+DaXαk DaXαk πi−DaXαk Fik], (25)

and


$${dp}^{0}=-\frac{\partial H_{0}^{\prime }}{\partial A_{0}}dt-\frac{\partial H_{1}^{\prime }}{\partial A_{0}}d{\overset{―}{A}}_{0}-\frac{\partial H_{2}^{\prime }}{\partial A_{0}}dA_{0}=-\frac{\partial H_{0}^{\prime }}{\partial A_{0}}dt=-\frac{\partial H_{c}}{\partial A_{0}}dt\;$$

dp0=−∫d3x[(A―i−DaXαi A0)(∂(Ai―−DaXαi A0)∂A0)−2a2(DaXαi A―i−DaXαi DaXαi A0)(∂(DaXαk A―k−DaXαi DaXαi A0)∂A0)]dt

dp0=[−DaXαi (A―i−DaXαi A0)−2a2DaXαk DaXαk (DaXαi A―i−DaXαi DaXαi A0)]dt (26)

Finally, for $\pi^{i}$ we have:


$$d\pi^{i}\;=-\frac{\partial H_{0}^{\prime }}{\partial {\overset{―}{A}}_{i}}dt-\frac{\partial H_{1}^{\prime }}{\partial {\overset{―}{A}}_{i}}d{\overset{―}{A}}_{0}-\frac{\partial H_{2}^{\prime }}{\partial {\overset{―}{A}}_{i}}dA_{0}=-\frac{\partial H_{0}^{\prime }}{\partial {\overset{―}{A}}_{i}}dt=-\frac{\partial H_{c}}{\partial {\overset{―}{A}}_{i}}dt$$

dπi=−∫d3x[pj∂A―j∂Ai+(A―j−DaXαj A0)(∂(A―j−DaXαj A0)∂A0)−2a2(DaXαj A―j+DaX2αA0)(∂(DaXαk A―k+DaX2αA0)∂A0)]dt

dπi=[−pj−(F0i)−2a2DaXαi DaXαk F0k]dt (27)

Equations (23), (24), and (25) are completely equivalent to equations (22), (23), and (26); as a result, equations (24) and (25) have the secondary limit that is absent from the total differential equations.

## 5. Fractional calculus applications and research suggestions

Fractional derivatives present two key advantages over classical derivatives: enhanced flexibility and non-local behavior. Their fractional order allows for a more adaptable fit to real-world data compared to classical derivatives. This study is aimed at scientists who are investigating fractional calculus and possible applications in the real world. It also provides recommendations for additional research into the generalized electrodynamic fields of Podolsky.

### 5.1. Fractional calculus application and research suggestions for podolskys equations

In 1936, Nathan Rosen and Boris Podolsky developed the Podolsky equations, which explain how an electromagnetic field behaves in a gravitational backdrop. Through the application of fractional calculus operators to generalize these equations, scientists can investigate the behavior of electromagnetic fields under gravitational conditions. Numerical solutions for fractional Podolsky’s equations can be obtained by applying numerical techniques such as spectral methods, finite difference, and finite element approaches. Stability and controllability of fractional Podolsky’s equations can be studied by treating them as fractional order systems. The study of electromagnetic field dynamics in quantum mechanical situations, especially in quantum electrodynamics, can also be done using fractional calculus.

### 5.2. Future directions and recommendations for further work

Podolsky’s fractional equations have garnered significant attention in recent years, demonstrating applications across various scientific and technical domains. Two key recommendations can advance this field of research.

First, it is essential to address the challenges posed by higher derivatives in equations of motion, as these can lead to unbounded energy states and negative probabilities, contradicting fundamental principles of physics. Solutions have been proposed, such as restricting theories to avoid unphysical scenarios and introducing auxiliary fields to eliminate problematic degrees of freedom. Despite these efforts, developing consistent and physically relevant higher-derivative theories remains a challenge. Further work in this area should focus on refining theoretical frameworks and exploring innovative strategies to tackle these issues while preserving the integrity of fundamental physics.

Second, Podolsky’s fractional equations facilitate a deeper understanding of fluid flow in porous materials, including rocks, soil, and membranes. Traditional equations often fail to provide accurate descriptions of fluid dynamics in these contexts. By employing fractional derivatives to capture long-range and non-local interactions, researchers can enhance predictions regarding porous structures and improve the modeling of materials with fractional-order characteristics, especially when memory effects are present. Further efforts should aim to drive the development and evaluation of novel materials with unique properties, potentially leading to advancements in applications such as energy storage systems and smart materials.

## 6. Comparison of results with other studies: Similarities and differences

This study compares our findings with the work of other researchers in the field of fractional electrodynamics. Our results regarding the Riemann-Liouville constraints in second-order Lagrangian models align significantly with research by [40], which highlights the effectiveness of fractional derivatives in addressing challenges related to non-local models. His recent work further explores the impact of these derivatives, demonstrating their value in capturing memory effects and long-range interactions, findings that are consistent with the focus of our study.

The work in [41] provided comparative insights into fractional-order models for capacitors, particularly emphasizing numerical strategies. Their analysis of the convergence of solutions in fractional-order systems parallels our numerical approach in electrodynamics. The robustness and accuracy of their numerical methods, particularly in time-varying problems, reflect similar strengths in our methodology, highlighting the broad applicability of fractional calculus across different domains. Furthermore, our extension of the Hamilton-Jacobi formalism aligns with the findings of [42]. In their work on time-fractional electrodynamics. Their research expands the Hamilton-Jacobi framework by incorporating fractional terms, which resonates with our approach to enhancing the understanding of the interplay between classical and fractional systems. Both studies contribute to a deeper comprehension of how fractional models can bridge gaps in traditional electrodynamics theories.

## 7. Conclusions

The canonical structure of generalized electrodynamics is established by two mathematical techniques: Dirac’s theory of confined systems and the Second-Order Lagrangian Fractional Model. These methods allow for the investigation of fractional dynamical variables, the construction of Hamiltonian constraints, and the precise deduction of gauge transformations. A thorough analysis of Podolsky’s electrodynamics is also provided, with an emphasis on the extended superposition principle and special relativity. In this study, we used Dirac’s theory to investigate Popolsky’s generalized electrodynamics. We found the Hamiltonian constraints and created a generator for second-order Lagrangian fractions. We focused on a specific system with second-order fractional differential equations to better understand the topic. The novelty of this work lies in the integration of fractional dynamics with established electrodynamic principles, offering fresh insights into the behavior of systems characterized by memory effects and non-local interactions. Our findings reveal that classical results are obtained when the fractional formulation is limited to derivatives of integer orders. This underscores the relevance of our approach, as it not only confirms previous theories but also extends them by providing a framework that accommodates a broader range of dynamical behaviors, thereby advancing the understanding of fractional electrodynamics beyond existing literature. While these mathematical techniques provide valuable insights, they also introduce significant challenges that must be addressed. One of the numerous challenges in the study was the complexity of second-order fractional derivatives, particularly the Riemann-Liouville derivative, which complicates modeling and solution derivation. The non-local nature of fractional calculus makes practical applications even more challenging. Despite improving Podolsky’s electrodynamics, the model’s complexity makes it unsuitable for standard engineering applications, requiring advanced numerical methods and posing the risk of non-physical solutions such as unbounded energy states.

## Supporting information

S1 File Appendix A. (DOCX)
