# Correction: Analyzing Riemann-Liouville constraints in second-order Lagrangian fractional electrodynamic models

**DOI:** 10.1371/journal.pone.0343049

**Published:** 2026-02-17

**Authors:** Yazen M. Alawaideh, Bashar M. Al-khamiseh, Isaac Kwasi Adu

The affiliation for the first author is incorrect. The correct affiliation is not indicated. Yazen M. Alawaideh is not affiliated with #1 but with: Applied Science Research Center, Applied Science Private University, Amman 11931, Jordan.
