# Supplementary material for: Analyzing Riemann-Liouville constraints in second-order Lagrangian fractional electrodynamic models
Source: PLoS One. 2025 May 27;20(5):e0320632. doi: 10.1371/journal.pone.0320632 (PMC12112156; doi:10.1371/journal.pone.0320632)
Supplement: S1 File — (DOCX) [file pone.0320632.s001.docx]

Appendix A

Podolsky's Electrostatics Generalized Using Special Relativity

Podolsky's electrostatics is a theory that extends classical electrostatics by incorporating the effects of special relativity. The idea was introduced by Boris Podolsky in the 1940s and is based on the concept of "generalized" Coulomb's law, which considers the finite size of charged particles.

In Podolsky's theory, the electrostatic potential is represented as a function of the point charge$Q$, the distance between the charges r, and$4\pi$ a constant. The electrostatic potential can be calculated using the formula:

$$v=\frac{Q}{4\pi}\left( \frac{1-e^{-rM}}{r} \right) (A1)$$

where$rM$ represents the distance at which the electrostatic potential is being calculated, and the function $e^{-rM}$ accounts for the decrease in potential as distance increases.

Using generalized Coulomb's law and the superposition theory, we can calculate the electric field $\vec{E}\left( \vec{r} \right)$ resulting from electrostatic charge density $\rho\left( \vec{r} \right)$. The electric field can be computed using the equation:

$$\vec{E}\left( \vec{r} \right)=\int d^{3}\acute{r}\frac{\rho\left( \vec{r} \right)}{4\pi}\left( \frac{1-e^{-RM}}{R^{2}}-\frac{Me^{-RM}}{R} \right)\frac{\vec{R}}{R} (A2)$$

According to the divergence theorem, we can infer that the electric field flux through a closed surface is proportional to the enclosed charge. This theorem plus a few algebraic operations allows us to find the differential equation for the electric field, which gives us the following:

$$(1-a^{2}\nabla^{2})\nabla.E⃗ (\vec{r} )=\rho(\vec{r} ) (A3)$$

where $a$ is a constant related to the size of the charged particles. We can also derive the equation for the curl of the electric field, which is given by

$$\nabla\times E ⃗(\vec{r})=0. (A4)$$

This equation indicates that the electrostatic, which means that the path taken by a charged particle has no effect on the work the field performs on it.

By adding the effects of special relativity, Podolsky’s electrostatics theory expands on classical electrostatics. The hypothesis is predicted on the idea of generalized Coulomb’s law, which considers charged particles finite size. As functions of point charge, the separation between the charges and a constant, the electrostatic potential and electric field are shown. The divergence and curl rules of vector calculus, as well as the conservation of energy, are all supported by the theory.
